# Ion-Selective Electrode for Nitrates Based on a Black PCV Membrane

**DOI:** 10.3390/molecules29153473

**Published:** 2024-07-25

**Authors:** Nikola Lenar, Martyna Drużyńska, Robert Piech, Beata Paczosa-Bator

**Affiliations:** Faculty of Materials Science and Ceramics, AGH University of Krakow, Mickiewicza 30, PL-30059 Krakow, Poland; nlenar@agh.edu.pl (N.L.); druzynska@student.agh.edu.pl (M.D.); rpiech@agh.edu.pl (R.P.)

**Keywords:** carbon nanomaterials, single-piece electrode, PVC membrane, nitrate sensor, potentiometry

## Abstract

Carbon nanomaterials were introduced into this research as modifiers for polymeric membranes for single-piece electrodes, and their properties were studied for the case of nitrate-selective sensors. The use of graphene, carbon black and carbon nanotubes is shown to significantly improve the potentiometric response, while no redox response was observed. The use of carbon nanomaterials results in a near-Nernstian response (54 mV/pNO_3_^−^) towards nitrate ions over a wide linear range (from 10^−1^ to 10^−6^ M NO_3_^−^). The results obtained by chronopotentiometry and electrochemical impedance spectroscopy reveal little resistance, and the capacitance parameter is as high as 0.9 mF (for graphene-based sensor). The high electrical capacity of electrodes results in the good stability of the potentiometric response and a low potential drift (0.065 mV/h). Introducing carbon nanomaterials into the polymetric membrane, instead of using them as separate layers, allows for the simplification of the sensors’ preparation procedure. With single-piece electrodes, one step of the procedure could be omitted, in comparison to the procedure for the preparation of solid-contact electrodes.

## 1. Introduction

Ion-selective electrodes (ISEs), over the decades of their development, have become a robust tool for ion analysis. Currently, potentiometry allows for the trace level measurements of micromolar activities, with a stable response offered by modern ISEs of various constructions. All-solid-state electrodes, characterized by the absence of any internal solutions, are considered as most promising in terms of their advantages in simple fabrication, easy miniaturization and great analytical properties [1,2,3]. The first ever solid sensors, named coated-wire electrodes, suffer from potential instability caused by a low double layer capacitance, a high charge transfer resistance, and also the formation of a thin aqueous layer at the membrane/metal interface [4,5,6]. The stability of the potentiometric response of all-solid-state sensors was improved thanks to the application of a redox-active, hydrophobic material of high surface area acting as a connector between an ionic and electronic conductor [7]. This idea was introduced as solid-contact electrode by Cadogan et al. in 1992 [8], with conducting polymer acting as an internal material.

Since that time, many different materials were implemented as solid-contact layers, allowing for the improvement of sensors’ properties, including the wide group of conducting polymers: poly(3-octylthiophene) (POT) [9], poly(aniline) (PANI) [10], poly(3,4-ethylene- 1,4-dioxythiophene) (PEDOT) [7], and PEDOT doped with other ions: PEDOT(PSS) and PEDOT(Cl) [7]; the group of molecular organic compounds, such as 7,7,8,8-tetracyanoquinodimethane (TCNQ) [11] and tetrathiafulvalene (TTF) and their salts [12]; the group of carbon nanomaterials: carbon nanotubes [13], carbon nanohorns [14], graphene [15], carbon black [16], three-dimensionally ordered macroporous (3DOM) carbon [17], fullerenes [18], mesoporous carbon colloidal CIM [19]; metal nanoparticles: gold nanoparticles [20], platinum [21], and nanoporous gold films [22]; and the group of metal oxides (MO_x_) including IrO_2_ [23], RuO_2_ [24], MoO_2_ [25], MnO_2_ [26], CuO [27], ZnO [27].

Despite the great success of solid-contact ISEs, this construction of electrodes requires an additional step to produce the intermediate solid-contact layer, which makes this type of electrode more complicated to prepare than the coated-wire electrode. In 1995, Bobacka et al. [10] reported an easier method to fabricate all-solid-state electrodes, named a single-piece electrode, by adding active material, e.g., conducting polymer directly into the sensing cocktail. Subsequently, other materials were introduced directly into the membrane, creating a group of single-piece electrodes of increasing interest [28,29,30,31,32,33].

Plasticized PVC is the most commonly used matrix for solvent polymeric membranes, since such material provides optimum physical properties and ensures the relatively high mobility of the membrane constituents [28]. In this work, we developed a nitrate-selective polymetric PVC-based membrane with dispersed carbon nanomaterials, including carbon black, graphene and carbon nanotubes. Those membranes were applied onto glassy carbon (GC) disk electrodes to create robust single-piece electrodes for nitrate ion determination.

## 2. Sensor Preparation

The procedure of single-piece electrode preparation was developed to be as fast and easy as possible. The introduction of an active material (carbon nanomaterial) directly into a membrane, instead of applying it as a separate layer, allowed for a shorter preparation time in reference to the preparation of solid-contact electrodes. The procedure began with the preparation of GC disk electrodes’ surfaces by polishing them with alumina slurries and rinsing with water and methanol.

The PVC-based membranes were prepared using membrane components (*w*/*w*%): polyvinyl chloride (33.2%), lipophilic salt (0.7%), plasticizer (65%) and ionophore (1.1%). After dissolving all chemicals in tetrahydrofuran, carbon nanomaterials were added to three separate vials with a nitrate-selective membrane. Each vial included 4% (*w*/*w*) of a different carbon nanomaterial: graphene, carbon black and carbon nanotubes. One vial was left without additives so a group of control electrodes could be prepared.

Membranes were casted onto a surface of GC electrodes using a drop casting technique, with the volume of 60 µL. Five items of electrodes representing each group were prepared: sensors with a graphene-modified membrane (GC/GR-NO_3_^−^ISM), sensors with a carbon black-modified membrane (GC/CB-NO_3_^−^ISM), sensors with a nanotube-modified membrane (GC/NT-NO_3_^−^ISM) and sensors with a non-modified membrane (GC/NO_3_^−^ISM).

**Figure 1:**
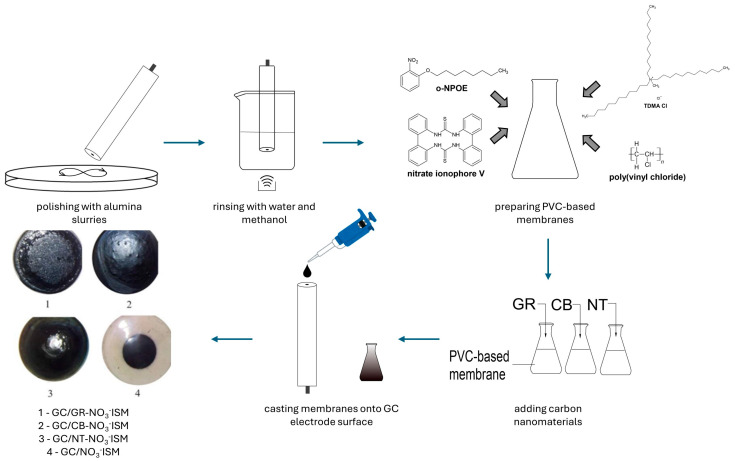
Schematic representation of the preparation procedure for the black PVC membrane sensors for nitrate determination.

The whole procedure lasted only a few minutes and required minimal use of chemicals. Also, what should be emphasized here is that only the basic laboratory equipment was used for this procedure. The schematic representation of the preparation procedure is presented in Figure 1.

## 3. Results

The results section can be divided into results obtained during the potentiometric measurements (analytical parameters), those obtained using chronopotentiometry and electrochemical impedance spectroscopy techniques (electrical parameters), and the SEM scans of modified nitrate-selective membranes.

### 3.1. Ionic Response

**Figure 2:**
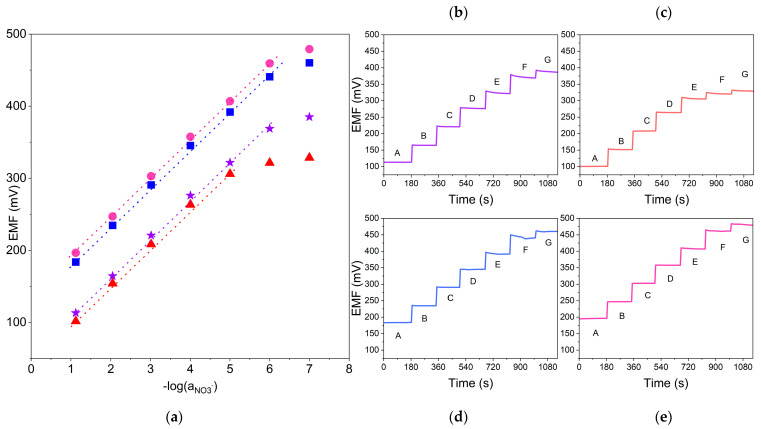
Exemplary potentiometric response towards nitrate ions recorded after 24 h of sensors’ conditioning in 10^−5^ M standard KNO_3_ solution for GC/GR-NO_3_^−^ISM (pink circle), GC/CB-NO_3_^−^ISM (blue square), GC/NT-NO_3_^−^ISM (purple star) and GC/NO_3_^−^ISM (red triangle) sensors. (**a**): calibration curve, (**b**–**e**): potential–time response recorded in A: 10^−1^ M B: 10^−2^ M C: 10^−3^ M D: 10^−4^ M E: 10^−5^ M F: 10^−6^ M G: 10^−7^ M nitrate ion standard solutions for each tested electrode.

Ionic response was examined using the potentiometry method. The sensors’ response towards nitrate ions was evaluated based on the parameters of calibration curves. Calibrations were conducted after 24, 48 and 72 h from the beginning of electrodes’ conditioning in 10^−5^ M standard KNO_3_ solution. The exemplary potentiometric response (the relationship between EMF and pNO_3_^−^ value) of the designed sensors (one item of each group) is presented in Figure 2.

The increase in EMF is proportional to the increase in the NO_3_^−^ activity value. The electrodes exhibit a near-Nernstian response towards the changes in activity of nitrate ions. The linear range of modified sensors covers the nitrate ions concentration range from 10^−1^ to 10^−6^ M, while the linear range of non-modified sensor covers values from 10^−1^ to 10^−5^ M.

### 3.2. Reproducibility

The comparison of electrodes’ parameters within each group provides information about the reproducibility of potentiometric response. The indicating factor of reproducibility was the standard deviation value, calculated based on the results obtained for each group (n = 5 items of electrodes within one group). The results are presented in Table 1. The measurements confirmed a good reproducibility of modified electrodes in comparison to non-modified ones, and that the registered potential was repetitive.

The addition of carbon nanomaterials to membranes causes better reproducibility compared to a non-modified electrode. Based on the standard deviation values, the group of electrodes modified with carbon black exhibit the highest reproducibility of the slope of calibration curve. The group of sensors with membranes modified with carbon nanotubes exhibit the highest reproducibility of the standard potential value. Within a group of non-modified electrodes, the lowest reproducibility is observed.

The addition of carbon nanomaterials significantly lowers the value of limit of detection (LoD). Electrodes modified with carbon nanotubes exhibit the lowest LoD of 2.31 × 10^−6^ M. Non-modified electrodes exhibit a much higher limit of detection in comparison to modified electrodes.

### 3.3. Potential Reversibility

**Figure 3:**
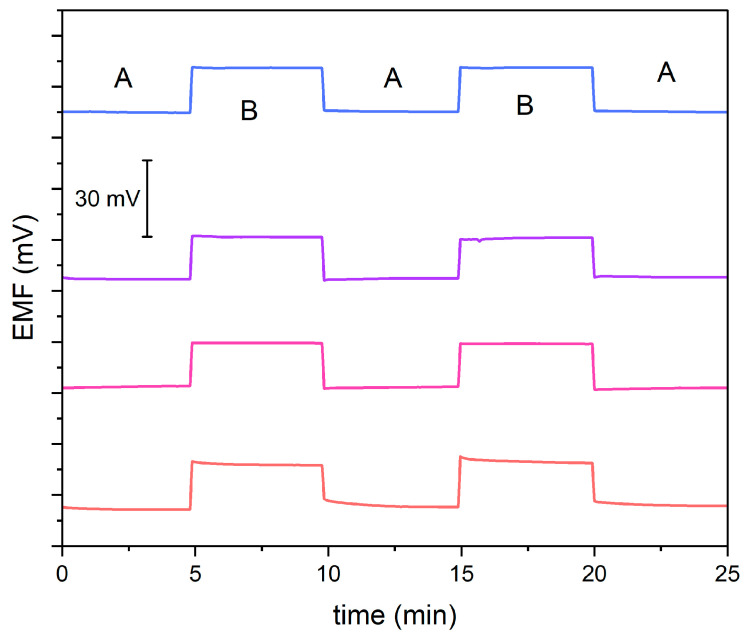
Reversibility test recorded in 10^−4.5^ M (A) and 10^−4^ M (B) standard KNO_3_ solution for GC/GR-NO_3_^−^ISM (pink line), GC/CB-NO_3_^−^ISM (blue line), GC/NT-NO_3_^−^ISM (purple line) and GC/NO_3_^−^ISM (red line) sensor.

The main goal of this experiment was to evaluate the reversibility of the potential and the stability of the potentiometric response after exchanging the analyzed solutions. For this purpose, using a 10^−4.5^ M KNO_3_ solution (A) and a 10^−4^ M KNO_3_ solution (B), the potential was measured alternately for 5 min, as presented in Figure 3. Modified electrodes exhibit stable and reversible potential. Slight changes in stability were observed in a non-modified electrode.

### 3.4. Redox Sensitivity

**Figure 4:**
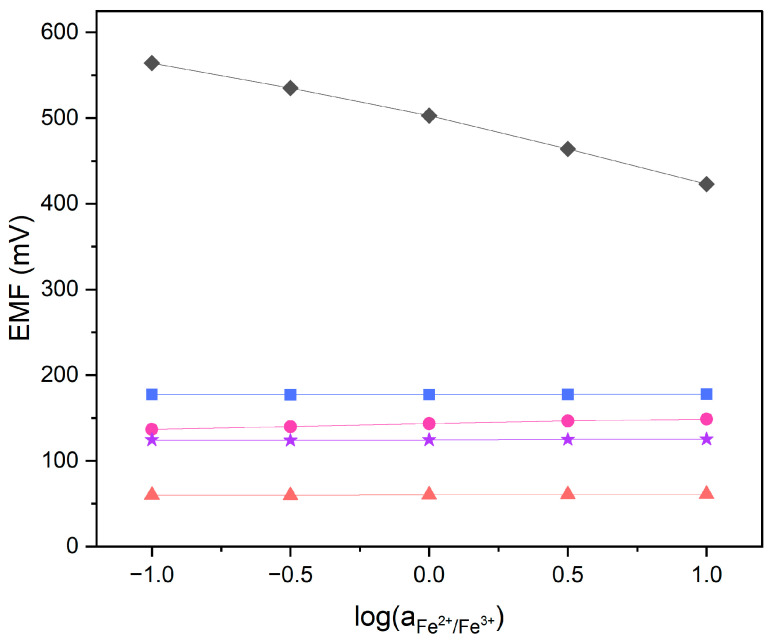
Redox test performed in FeCl_2_/FeCl_3_ solutions for GC/GR-NO_3_^−^ISM (pink circle), GC/CB-NO_3_^−^ISM (blue square), GC/NT-NO_3_^−^ISM (purple star), GC/NO_3_^−^ISM (red triangle) sensor and glassy carbon disk electrode (black diamond).

Carbon nanomaterials, which are used in this research as membrane modifiers, are known to be redox-sensitive substances. The redox sensitivity test was conducted in order to confirm that obtained nitrate-selective membranes modified with graphene, carbon black and carbon nanotubes are not sensitive to changing redox conditions. Potential measurements were performed in solutions containing constant amounts of a FeCl_2_ and FeCl_3_ redox, coupled with the log of a Fe^2+^/Fe^3+^ ratio equal to −1, −0.5, 0, 0.5 and 1. No redox response was registered in the electrodes with modified membranes. The GC disk electrode exhibited a near-Nernstian response towards the change in Fe^2+^/Fe^3+^ activity. Graphene, carbon black and carbon nanotubes were expected to exhibit redox response, yet the membrane was shown to act as an electronic insulator. The examined sensors are not redox sensitive, as presented in Figure 4.

### 3.5. Potential Stability

The stability of the potentiometric response was tested during the long-time (24 h) measurement. One item of each group of designed electrodes was tested in the 10^−5^ M standard nitrate ion solution during the potentiometric measurement, versus the reference Ag/AgCl electrode. The potential stability of the sensors was assessed using the potential drift parameter, which is calculated as potential difference/time ratio. The most stable potentiometric response is attributed to the graphene-modified electrode (with 0.065 mV/h potential drift); for the nanotube- and carbon black-modified electrodes, the potential drift was 0.082 mV/h and 0.087 mV/h, respectively, and the most significant potential drift was observed for the non-modified electrode (0.85 mV/h).

As found in the literature, for electrodes with polymeric membrane, without any modifications, the potential drift is usually equal to ~1 mV/h, and, thanks to the application of additional high-capacity materials, the potential drift value decreases. The obtained values of electrical capacitance parameters for the carbon nanomaterial-based electrodes in line with the results presented in the literature for nitrate-selective electrodes. Niemiec et al. [34] presented a paste NO_3_^−^-selective electrode with carbon black, and obtained potential drift values of 0.12 mV/h, which are considerably higher in comparison to the value presented in this paper for carbon black-modified electrodes (0.082 mV/h).

### 3.6. Water Layer Test

**Figure 5:**
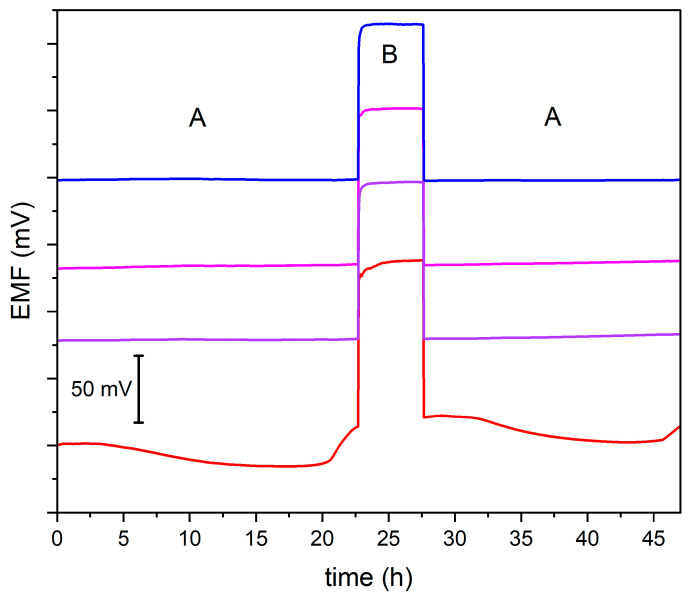
Water layer test performed in 10^−2^ M standard KNO_3_ (A) and 10^−2^ M standard KCl (B) solution for GC/GR-NO_3_^−^ISM (pink line), GC/CB-NO_3_^−^ISM (blue line), GC/NT-NO_3_^−^ISM (purple line) and GC/NO_3_^−^ISM (red line) sensor.

A water layer test was conducted to assess if carbon nanomaterials, used as modifiers added directly to an ion-selective membrane, prevent the formation of a water layer. The potential was registered for 24 h in a KNO_3_ solution (A) of 10^−2^ M concentration. Next measurement was performed for 5 h in a KCl solution (B) of 10^−2^ M concentration. Then, for another 20 h, measurements were performed using solution A. Carbon nanomaterial-modified electrodes exhibit stable potential, while significant potential instability was observed for non-modified electrode (Figure 5). It can be concluded that modified electrodes inhibit the formation of a water layer.

### 3.7. Electrical Properties

Chronopotentiometry measurements were performed to examine the influence of the current flow through the measuring cell on the stability of the electrode response signal. Electrical capacitance parameter (C) was calculated using the following equation:

(1) $$C=I\cdot \frac{dt}{dE_{dc}}$$

where I stands for current, and $\frac{dt}{dE_{dc}}$ stands for potential drift. The resistance (R) was calculated using current (I) and potential jump ($\Delta E_{dc})$ with the following equation.

(2) $$R=\frac{I}{\Delta E_{dc}}$$

Modified electrodes exhibit much higher electrical capacity and lower resistance in contrast to non-modified electrodes.

**Figure 6:**
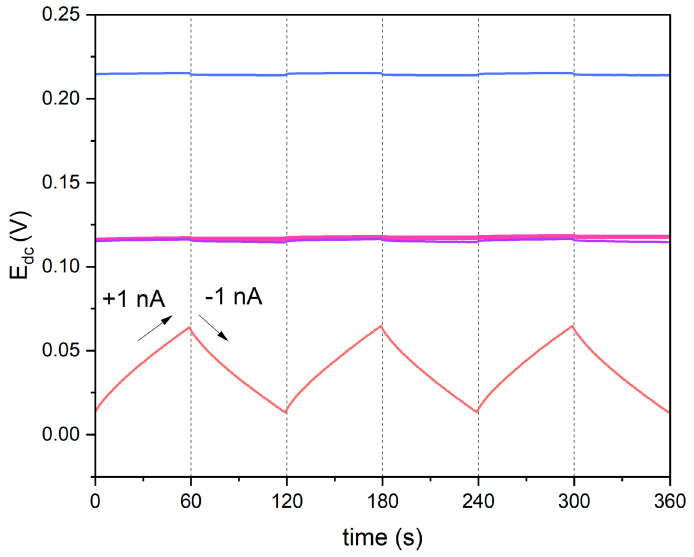
Chronopotentiograms recorded in 10^−1^ M standard KNO_3_ solution for GC/GR-NO_3_^−^ISM (pink line), GC/CB-NO_3_^−^ISM (blue line), GC/NT-NO_3_^−^ISM (purple line) and GC/NO_3_^−^ISM (red line) sensor, with the forced current flow of 1 nA.

During the measurement, six steps were recorded, which is shown in Figure 6. The carbon nanomaterial-modified electrodes exhibited a lower potential response to the current flow in comparison to non-modified electrodes. The results (electrical capacitance and resistance parameter values) are presented in Table 2, together with the values obtained with the EIS technique.

**Figure 7:**
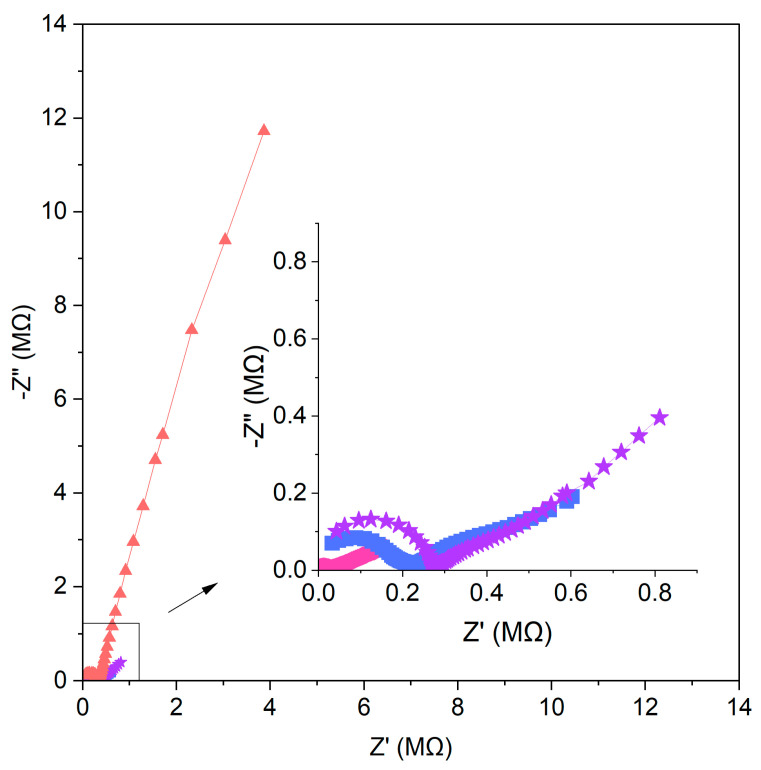
Nyquist plots recorded in 10^−1^ M standard KNO_3_ solution for GC/GR-NO_3_^−^ISM (pink circle), GC/CB-NO_3_^−^ISM (blue square), GC/NT-NO_3_^−^ISM (purple star) and GC/NO_3_^−^ISM (red triangle) sensor.

Electrochemical impedance spectroscopy measurements were carried out to calculate the resistance and electrical capacity of the electrodes. The electrical capacitance parameter (C) was calculated using the following equation:

(3) $$C=\left|\frac{1}{2\pi \cdot f\cdot Z\prime \prime }\right|$$

where *f* stands for frequency and *Z*″ stands for reactance. Resistance value R_ct_ (charge transfer resistance) was extracted from the Nyquist plot shown in Figure 7. Figure 7 shows the impedance spectra for one item representing each group of electrodes.

The low-frequency part of the EIS for non-modified electrodes showed a semicircle arising from a small capacitance with a high resistance. The addition of carbon nanomaterials as modifiers change the value of the electrical capacity and resistance. The semicircles obtained for carbon nanomaterial-modified sensors are smaller, with lower resistance and higher electrical capacity. The values of the capacitance and resistance for the designed sensors are presented in Table 2.

The results received from chronopotentiometry and the EIS measurements vary from each other, as both methods are based on different theoretical fundamentals. Overall, the relation between results is maintained. The highest value of electrical capacity and the lowest value of resistance from both measurements can be attributed to the group of graphene-based electrodes.

The high electrical capacity of electrodes ensures stable potentiometric response over time, short response time and insensitivity to the perturbations that may occur during the course of measurement (e.g., power fluctuations). Low resistance is a key factor to ensure ion-to-electron transduction processes between the electronic and ionic conductor within an electrode. Facilitated thanks to the presence of carbon nanomaterials, the charge transfer processes ensure enhanced analytical properties and improved performance of an electrode [7]. Taking this into consideration, when designing new potentiometric sensors, it is desirable to obtain sensors of the highest electrical capacity and the lowest resistance possible.

The values of electrical capacitance attributed to the designed single-piece electrodes are in line with the values presented in the literature for solid-contact electrodes (SC-ISEs). For nanotube-modified ISEs, the electrical capacitance parameter evaluated with the chronopotentiometry method is equal to 88 µF, while Crespo et al. reported the value of 60 µF [13] for the nanotube-contacted SC-ISEs. Also, in the case of carbon black, the electrical capacitance parameter is higher for the single-piece carbon black-modified sensor (375 µF), than for the SC-ISE (51 µF as reported by Paczosa-Bator [16]). The highest electrical capacity was observed for graphene-modified single-piece sensors (931 µF). The value obtained for the presented type of sensors is ten times higher in comparison to the graphene-contacted SC-ISEs (91 µF) as reported in a paper published by Li et al. [35].

### 3.8. Microstructure

**Figure 8:**
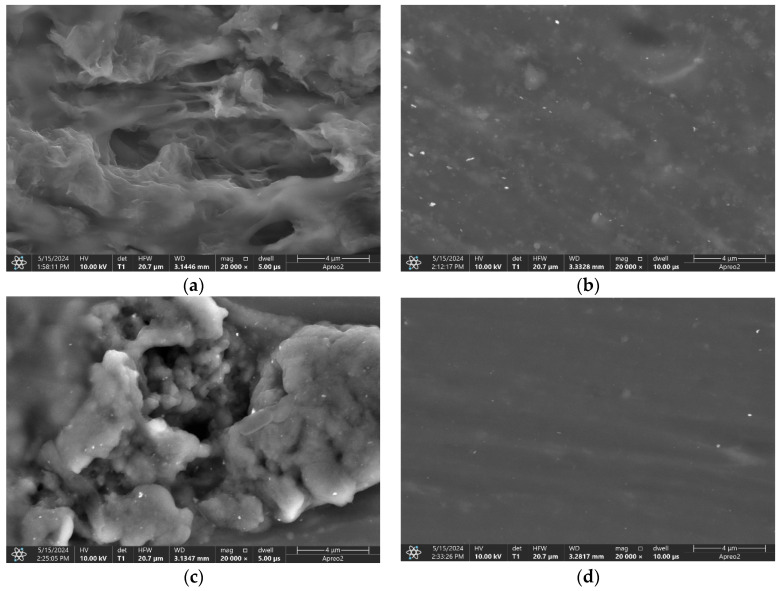
SEM scans of cross-sections of PVC membranes; (**a**) membrane modified with graphene, (**b**) membrane modified with carbon black, (**c**) membrane modified with carbon nanotubes, (**d**) non-modified membrane.

The cross- section SEM images present the morphology of the membranes (modified and non-modified) casted on the GC electrodes. Figure 8a shows the graphene-modified membrane’s morphology, Figure 8b presents the carbon black-modified membrane, and Figure 8c shows the carbon nanotube-modified polymeric PVC membrane. For comparison, Figure 8d presents the non-modified polymeric membrane.

Differences in the membranes’ morphologies were observed. Carbon black nanoparticles form agglomerates, while CNTs and graphene are diffused evenly in a polymer.

## 4. Materials and Methods

The utilized chemicals included those used for the design of single-piece electrodes and those necessary for the conducted tests and measurements.

The membrane components included: polyvinyl chloride of high molecular weight, lipophilic salt Tridodecylmethylammonium chloride (TDMA Cl), plasticizer 2-Nitrophenyl octyl ether (o-NPOE) and nitrate ionophore V. All components were dissolved in tetrahydrofuran (THF). All membrane components and a solvent were purchased from Sigma Aldrich, St. Louis, MO, USA. Carbon materials (CM), used as additives to membranes, including short single-walled carbon nanotubes, single layer graphene (GR), and carbon black (CB), were obtained from the Nanostructured & Amorphous Materials, Inc. (Houston, TX, USA), ACS Material (Pasadena, CA, USA), and 3D-nano, Krakow, Poland, respectively.

As a standard nitrate ion solution, potassium nitrate aqueous solutions were used. Potassium nitrate was purchased from POCH (Gliwice, Poland). The standard NO_3_^−^ ion solutions were used for all the conducted measurements, including potentiometry, electrochemical impedance spectroscopy, and chronopotentiometry. For the additional examination and validation of designed sensors, ferric(II) chloride and ferric(III) chloride and potassium chloride were used (all purchased from POCH).

For the preparation of aqueous solutions, distilled and deionized water was used. All chemicals were used as received, without any further purification.

The potentiometry method was the main electrochemical technique used for the examination and validation of designed sensors. Calibration curves, long time measurements and other tests were performed using a 16-channel mV-meter (Lawson Labs, Inc., Malvern, PA, USA). The potentiometric response towards nitrate ions was examined in the standard KNO_3_ solutions of 10^−7^ to 10^−1^ M concentration. Potentiometric measurements were conducted versus the reference electrode—Ag/AgCl electrode with 3 M KCl solution (6.0733.100 Metrohm, Herisau, Switzerland) and in the presence of an auxiliary electrode (platinum wire).

The chronopotentiometry and electrochemical impedance spectroscopy measurements were carried out with the use of an Autolab General Purpose Electrochemical System (AUT302N.FRA2-AUTOLAB, Metrohm Autolab, Barendrecht, The Netherlands) with NOVA 2.1. software. Designed single-piece ISEs were tested as working electrodes in a three-electrode cell with a reference electrode Ag/AgCl electrode with 3 M KCl solution (6.0733.100 Metrohm, Herisau, Switzerland), and in the presence of an auxiliary electrode (glassy carbon electrode). Chronopotentiometric tests were conducted in the standard KNO_3_ solution of 10^−1^ M concentration. A constant current of +1 nA was applied to the working electrode for 60 s, followed by a −1 nA current for another 60 s, according to the procedure proposed by Bobacka [7]. Nyquist plots were recorded during the measurement of electrochemical spectroscopy impedance, performed in the standard KNO_3_ solution of 10–1 M concentration. The impedance spectra were obtained by applying a frequency from 100 kHz to 0.01 Hz using an AC amplitude of 10 mV superimposed on 0.15 V versus reference electrode.

The microstructure of nitrate-selective membranes was examined using a scanning electron microscope. SEM scans were collected using Scanning Electron Microscope-Apreo 2 (Thermo Scientific, Waltham, MA, USA).

## 5. Conclusions

Single-piece electrodes added to ion-selective membranes with carbon nanomaterials used as modifiers exhibit excellent analytical properties, comparable to solid-contact electrodes with carbon nanomaterials used as separate layers. The presented type of sensors allows for the simplification of the preparation procedure of sensors, as one step of the layer preparation can be omitted compared to solid-contact sensors. The preparation procedure is as short as a few minutes, and carbon nanomaterial-modified nitrate selective electrodes can be obtained using basic laboratory equipment with the minimal use of chemicals. The SEM scans proved that the distribution of carbon nanomaterials within polymeric membranes is equal; therefore, it can be concluded that proposed preparation procedure is sufficient.

The electrical properties of electrodes (evaluated with chronopotentiometry and electrochemical impedance spectroscopy) determine their analytical performance. For the graphene-modified sensor, the drift of potentiometric response was the slightest, which results from the significant value of electrical capacity that is attributed to these types of electrodes. The calibration curves obtained during potentiometric measurements confirm that sensors modified with carbon nanomaterials exhibit stable, repetitive, near-Nernstian potentiometric response in a concentration range between 10^−1^ to 10^−6^ M of nitrate ions. Despite the redox activity attributed to the carbon nanomaterials, the polymeric membrane single-piece sensors do not show any response towards changing redox conditions.

The research shows that application of carbon nanomaterials as additives to a polymeric membrane prevents the formation of water layer, which significantly contributes to the improvement in the potential stability of sensors.

## Figures and Tables

**Table 1:** Analytical parameters of designed carbon nanomaterial-based nitrate-selective electrodes: graphene, carbon black, carbon nanotube-modified and non-modified sensors compared within each group, together with standard deviation values (n = 5 items).

Electrode Symbol	Sensitivity S ± SD [mV]	Standard Potential E^0^ ± SD [mV]	Linear Range [M]	Limit of Detection [M]
GC/GR-NO_3_^−^ISM	−54.32 ± 0.39	141 ± 6	10^−1^–10^−6^	2.63 × 10^−6^
GC/CB-NO_3_^−^ISM	−54.22 ± 0.09	106 ± 3	10^−1^–10^−6^	2.95 × 10^−6^
GC/NT-NO_3_^−^ISM	−54.15 ± 0.18	37 ± 2	10^−1^–10^−6^	2.31 × 10^−6^
GC/NO_3_^−^ISM	−54.41 ± 1.02	−19 ± 7	10^−1^–10^−5^	2.34 × 10^−5^

**Table 2:** Electrical parameters of designed carbon nanomaterial-modified nitrate-selective sensors evaluated using chronopotentiometry and electrochemical impedance spectroscopy techniques (n = 3 items).

Electrode Symbol	Chronopotentiometry		Electrochemical Impedance Spectroscopy	
	Resistance [kΩ]	Electrical Capacity [µF]	Resistance [kΩ]	Electrical Capacity [µF]
GC/GR-NO_3_^−^ISM	46 ± 2	931 ± 2	28 ± 2	262 ± 4
GC/CB-NO_3_^−^ISM	375 ± 3	211 ± 3	220 ± 2	83 ± 2
GC/NT-NO_3_^−^ISM	331 ± 2	88 ± 1	276 ± 3	40 ± 2
GC/NO_3_^−^ISM	915 ± 8	1.19 ± 0.03	323 ± 7	1.35 ± 0.09

## Data Availability

Data are contained within the article.
